# Risk factors for prolonged mechanical ventilation in critically ill patients with influenza-related acute respiratory distress syndrome

**DOI:** 10.1186/s12931-023-02648-3

**Published:** 2024-01-04

**Authors:** Pai-Chi Hsu, Yi-Tsung Lin, Kuo-Chin Kao, Chung-Kan Peng, Chau-Chyun Sheu, Shinn-Jye Liang, Ming-Cheng Chan, Hao-Chien Wang, Yu-Mu Chen, Wei-Chih Chen, Kuang-Yao Yang, TSIRC (Taiwan Severe Influenza Research Consortium), Han-Chung Hu, Wann-Cherng Perng, Ming-Ju Tsai, Chieh-Liang Wu, Ying-Chun Chien, Wen-Feng Fang

**Affiliations:** 1 Institute of Emergency and Critical Care Medicine, National Yang Ming Chiao Tung University, Taipei, Taiwan; 2 Department of Respiratory Therapy, Sijhih Cathay General Hospital, New Taipei, Taiwan; 3 School of Medicine, College of Medicine, National Yang Ming Chiao Tung University, Taipei, 112 Taiwan; 4 Division of Infectious Diseases, Department of Medicine, Taipei Veterans General Hospital, Taipei, Taiwan; 5 Department of Thoracic Medicine, Chang Gung Memorial Hospital, Taoyuan, Taiwan; 6 Division of Pulmonary and Critical Care Medicine, Department of Internal Medicine, Tri-Service General Hospital, Taipei, Taiwan; 7 Division of Pulmonary and Critical Care Medicine, Kaohsiung Medical University Hospital, Kaohsiung, Taiwan; 8 Division of Pulmonary and Critical Care, Department of Internal Medicine, China Medical University Hospital, Taichung, Taiwan; 9 Department of Critical Care Medicine, Taichung Veterans General Hospital, Taichung, Taiwan; 10 Division of Chest Medicine, Department of Internal Medicine, National Taiwan University Hospital, Taipei, Taiwan; 11 Division of Pulmonary and Critical Care Medicine, Department of Internal Medicine, Chang Gung Memorial Hospital, Kaohsiung, Taiwan; 12 Department of Chest Medicine, Taipei Veterans General Hospital, # 201 Sec. 2, Shih-Pai Road, Taipei, 11217 Taiwan; 13 Cancer Progression Research Center, National Yang Ming Chiao Tung University, Taipei, Taiwan

## Abstract

**Background:**

Patients with influenza-related acute respiratory distress syndrome (ARDS) are critically ill and require mechanical ventilation (MV) support. Prolonged mechanical ventilation (PMV) is often seen in these cases and the optimal management strategy is not established. This study aimed to investigate risk factors for PMV and factors related to weaning failure in these patients.

**Methods:**

This retrospective cohort study was conducted by eight medical centers in Taiwan. All patients in the intensive care unit with virology-proven influenza-related ARDS requiring invasive MV from January 1 to March 31, 2016, were included. Demographic data, critical illness data and clinical outcomes were collected and analyzed. PMV is defined as mechanical ventilation use for more than 21 days.

**Results:**

There were 263 patients with influenza-related ARDS requiring invasive MV enrolled during the study period. Seventy-eight patients had PMV. The final weaning rate was 68.8% during 60 days of observation. The mortality rate in PMV group was 39.7%. Risk factors for PMV were body mass index (BMI) > 25 (kg/m^2^) [odds ratio (OR) 2.087; 95% confidence interval (CI) 1.006–4.329], extracorporeal membrane oxygenation (ECMO) use (OR 6.181; 95% CI 2.338–16.336), combined bacterial pneumonia (OR 4.115; 95% CI 2.002–8.456) and neuromuscular blockade use over 48 h (OR 2.8; 95% CI 1.334–5.879). In addition, risk factors for weaning failure in PMV patients were ECMO (OR 5.05; 95% CI 1.75–14.58) use and bacteremia (OR 3.91; 95% CI 1.20–12.69).

**Conclusions:**

Patients with influenza-related ARDS and PMV have a high mortality rate.

Risk factors for PMV include BMI > 25, ECMO use, combined bacterial pneumonia and neuromuscular blockade use over 48 h. In addition, ECMO use and bacteremia predict unsuccessful weaning in PMV patients.

**Supplementary Information:**

The online version contains supplementary material available at 10.1186/s12931-023-02648-3.

## Introduction

The advancement of high-quality critical care has saved numerous lives worldwide, allowing patients to survive; however, certain patients experience incomplete recovery, necessitating prolonged mechanical ventilation (PMV) [1]. PMV is defined as mechanical ventilation lasting 21 days or more for a minimum of 6 h daily [2]. The global population of PMV patients continues to rise, placing a burden on health care systems.

In a meta-analysis concerning post-acute care hospitals in the United States and other countries, the success rate of ventilator liberation for patients with a duration of mechanical ventilation (MV) exceeding 14 days was 47 and 63%, respectively. However, the one-year mortality rates were 73 and 47%, respectively. This study suggests that nearly half of the patients who are discharged alive from these facilities do not survive beyond one year [3].

Influenza infection can lead to severe pulmonary complications, including acute respiratory distress syndrome (ARDS) and respiratory failure [4]. Most of the related fatal cases involve young individuals who were previously healthy and without any underlying complications. [5]. Although patients with ARDS might improve rapidly, some patients had substantial injury over respiratory and other vital organs, leading to prolonged mechanical ventilation times and increased mortality rates [6, 7].

To date, most published studies on influenza-related ARDS have primarily focused on investigating risk factors associated with hospital mortality. However, there is limited information available regarding risk factors for PMV in ARDS. The primary objective of this study was to investigate risk factors for PMV in critically ill patients with influenza. The secondary objective was to evaluate risk factors associated with unsuccessful weaning among PMV patients.

## Methods

### Study design

This is a retrospective study that analyzed cohorts in Taiwan Severe Influenza Research Consortium (TSIRC). The study received approval from the institutional review boards of all participating hospitals (Taipei Veterans General Hospital, 2016–05-020CC; Taichung Veterans General Hospital, CE16093A; National Taiwan University Hospital, 201605036RIND; Tri-Service General Hospital, 1–105-05–086; Chang Gung Memorial Hospital, 201600988B0; China Medical University Hospital, 105-REC2-053(FR); Kaohsiung Medical University Hospital, KUMHIRB-E(I)-20170097; Kaohsiung Chang Gung Memorial Hospital, 201600988B0). Given that all patient information during the data recording period was anonymized and deidentified, informed consent was not needed.

The data collection period for this study spans January 1, 2016, to March 31, 2016. Patients diagnosed with ARDS attributed to influenza and requiring admission to the intensive care unit during this timeframe were included in the study. The inclusion criterion was patients diagnosed with influenza-associated ARDS who received invasive mechanical ventilation.

The severity of ARDS was classified according to the Berlin definition, which involves acute onset of respiratory distress within one week, radiographic confirmation of bilateral diffuse opacities, absence of evidence of heart failure as the primary cause of pulmonary edema, and arterial partial pressure of oxygen/fraction of inspired oxygen ratio < 300, with positive end-expiratory pressure ≥ 5 cm H2O[8]. We collected data for patients with influenza-induced ARDS requiring mechanical ventilation support while excluding those under 18 years old.

The initial cohort of 263 individuals was used for calculating overall ventilator weaning rates. Patients who died before the 21st day of mechanical ventilation were excluded. Patients in the PMV cohort remained on a ventilator wean plan beyond the 21st day of ventilation and constituted the group with unsuccessful weaning from ventilatory support post PMV.

### Data collection

We recorded demographic data, including sex, age, body mass index, and comorbidities, as well as clinical data during the intensive care unit stay, such as laboratory results and APACHE II severity scores [9]. Key invasive treatment measures and events and their timing following the onset of influenza-induced ARDS were analyzed, including ECMO, prone positioning, renal replacement therapy, vasopressor use, sedatives, neuromuscular blockers, and steroids. The occurrence and timing of bacterial pneumonia and bacteremia were also documented.

### Treatment outcome assessment

The main treatment outcomes assessed in this study were ventilator weaning rate, length of stay in the intensive care unit, hospital length of stay, duration of mechanical ventilation, and successful weaning from the ventilator or weaning failure.

The primary outcome of interest was PMV, as defined as invasive mechanical ventilation exceeding 21 days. In the PMV group, reintubation or death within 48 h or the need for mechanical ventilation at discharge was considered unsuccessful weaning from ventilation.

The primary objective of this study was to investigate risk factors for PMV in critically ill patients with influenza, with a secondary aim to evaluate factors associated with failure to wean from ventilation post PMV.

### Statistical analysis

Results are presented as means ± standard deviations, medians with interquartile ranges, or percentages. Pearson’s χ2 test or Fisher's exact test was used to compare categorical variables. The normality of continuous variables was assessed using the Kolmogorov‒Smirnov and Shapiro‒Wilk tests. Independent sample t tests or *Mann‒Whitney U* tests were used to compare differences between groups for continuous variables, depending on the distribution’s normality. Comparison of categorical variables was performed using exact tests.

Model building was carried out through initial screening using univariate analysis with a threshold of p < 0.1, followed by enter selection based on variable choice, with an entry criterion of 0.05. Both univariate and multivariate binary logistic regression analyses were conducted to identify variables showing significant differences between the two groups and to determine independent predictors of PMV. Odds ratios (ORs) and 95% confidence intervals (CIs) were calculated.

All p values were two-tailed, and p < 0.05 was considered significant. Forest plots were employed to visually depict effect sizes, with odds ratios reported along with their 95% confidence intervals. Finally, Kaplan‒Meier survival curves were plotted for each factor in multivariate models, and comparisons were made using the log-rank test.

The analyses were conducted using MedCalc version 19.2.5 (MedCalc Software Ltd, Ostend, Belgium) and IBM SPSS Statistics for Windows/Macintosh, Version 24.0 (IBM Corp., Armonk, NY, USA).

## Results

**Fig. 1:**
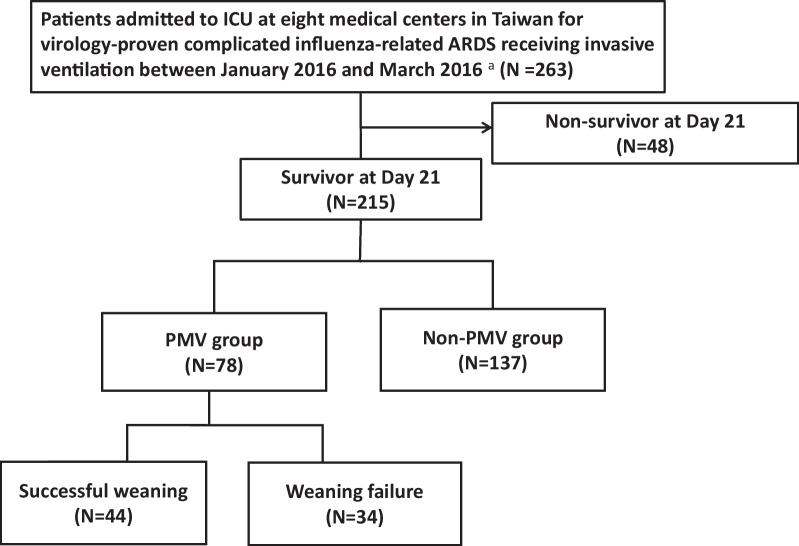
Flow chart of the study. **a** Virology-proven methods include the rapid influenza diagnostic test, reverse transcription-polymerase chain reaction and virus culture. *ARDS* acute respiratory distress syndrome; *ICU* intensive care unit; *MV* mechanical ventilator

During the course of the study, 263 patients with influenza-induced acute respiratory distress syndrome (ARDS) were included, with exclusion criteria being patients who by 21 post initiation of mechanical ventilation 48 patients had died. Forty-eight patients (18.3%) died up to day 21. The hospital mortality for the whole group of patients was 30.4% and 39.7% of patients requiring PMV eventually died in hospital before discharge. Ultimately, 215 patients met the criteria and were included in the analysis of PMV. Among them, 78 patients who underwent PMV and subsequently underwent risk assessment for inability to be weaned from the ventilator were further evaluated (Fig. 1). In our cohorts, all ECMO patients received sedatives and neuromuscular blockage agents. The PMV group presented higher BMI and APACHE II score, and a higher frequency of ECMO, bacterial pneumonia, bacteremia, neuromuscular blockade for more than 48 h, and vasopressor usage when compared with non-PMV group. Also, PMV group has worse outcome, including longer MV days, longer ICU stay, longer hospital days and higher in-hospital mortality (Table 1).

**Table 1:** Characteristics of the 215 subjects with influenza-related ARDS

	All patients	Prolonged mechanical ventilation (PMV)			Weaning in PMV		
Characteristics		Yes	No	*p* Value	Success	Failure	*p* Value
	(n = 215)	(n = 78)	(n = 137)		(n = 44)	(n = 34)	
Baseline data							
Age (years)	59.27(± 14.48)	58.22(± 11.74)	59.88(± 15.83)	0.38	59.25(± 12.12)	56.88(± 11.27)	038
Male sex	134(62.3%)	50(64.1%)	84(61.3%)	0.69	31(70.5%)	19(55.9%)	0.18
Body mass index BMI (kg/m2)	25.67(± 5.78)	26.84(± 4.77)	25.53(± 5.70)	0.087	26.65(± 4.19)	27.09(± 5.48)	0.68
BMI > 25 (kg/m2)	124(57.7%)	56(71.8%)	68(49.6%)^**^	0.002	23(67.6%)	33(75%)	0.47
Malignancy	26(12.1%)	12(15.4%)	14(10.2%)	0.26	7(15.9%)	5(14.7%)	0.88
Type II diabetes mellitus	63(29.3%)	22(28.2%)	41(29.9%)	0.79	11 (25%)	11(32.4%)	0.47
Cerebrovascular disease	14(6.5%)	4(5.1%)	10(7.3%)	0.54	4	0	0.13
Liver disease	22(10.2%)	9(11.5%)	13(9.5%)	0.63	4(11.8%)	5(11.4%)	1.0
Cardiac disease	25(11.6%)	5(6.4%)	20(14.6%)	0.072	3(8.8%)	2(4.5%)	0.65
Hypertension	93(43.3%)	35(44.9%)	58(42.3%)	0.72	19(43.2%)	16(47.1%)	0.73
Immunosuppressant^b^ use before influenza infection	10(4.7%)	5(6.4%)	5(3.6%)	0.5	1(2.3%)	3(8.8%)	0.31
Autoimmune disease	14(6.5%)	6(7.7%)	8(5.8%)	0.60	2(4.5%)	4(11.8%)	0.40
End-stage renal disease	14(6.5%)	7(9.0%)	7(5.1%)	0.27	4(9.1%)	3(8.8%)	1.0
Severity scores							
APACHE II score	22.59(± 8.13)	24.14(± 8.52)	21.7(± 7.8^)*^	0.034	23.30 ± 8.74	25.24(± 8.23)	0.32
ARDS ^a^ Severity				0.18			0.76
Severe	122(56.7%)	49(62.8%)	73(53.3%)		27(61.4%)	22(64.7%)	
Mild to moderate	93(43.3%)	29(37.2%)	64(46.7%)		17(38.6%)	12(35.3%)	
Treatments and clinical outcome							
Prone	49(22.8%)	23(29.5%)	26(19%)	0.08	14(31.8%)	9(26.5%)	0.61
ECMO	34(15.8%)	26(33.3%)	8(5.8%)^**^	< 0.01	8(18.2%)	18(52.9%)^**^	< 0.01
Combined with bacterial pneumonia onset before D21	67(31.2%)	39(50%)	28(20.4%)^**^	< 0.01	26(59.1%)	22(64.7%)	0.61
Bacteremia onset before D21	34(15.8%)	19(24.4%)	15(10.9%)^**^	0.01	6(13.6%)	13(38.2%)^*^	0.012
Steroid user	127(59.1%)	46(59%)	81(59.1%)	0.98	23(52.3%)	23(67.6%)	0.17
Sedation	159(74%)	60(76.9%)	99(72.3%)	0.45	32(72.7%)	28(82.4%)	0.32
Neuromuscular blockade > 48 h	119(55.3%)	59(75.6%)	60(43.8%)^**^^*^	< 0.001	32(72.7%)	27(79.4%)	0.5
Need for vasopressor agents	103(47.9%)	49(62.8%)	54(39.4%)^**^^*^	< 0.01	25(56.8%)	24(70.6%)	0.21
Renal replacement therapy ^c^	19(8.8%)	11(14.1%)	8(5.8%)^*^	0.04	6(13.6%)	5(14.7%)	1.0
Ventilator-duration (days)	21.18(± 17.74)	39.24(± 17.77)	10.90(± 4.77)^**^^*^	< 0.001	36.12(± 14.60)	43.29(± 20.72)	0.09
ICU stay (days)	22.32(± 18)	38.18(± 20.65)	13.22(± 6.24)^**^^*^	< 0.001	36(± 19.22)	41(± 22.34)	0.29
Hospital-stay (days)	37.59(± 27.38)	54.12(± 32.73)	28.11(± 18)^**^^*^	< 0.001	58.90(± 29.70)	47.92(± 35.77)	0.14
In hospital Mortality	42(19.5%)	31(39.7%)	11(8%)^**^^*^	< 0.001	5(11.4%)	26(76.5%)^***^	< 0.001

Data are presented as the mean ± standard deviation and number (%)

*APACHE II* Acute Physiology and Chronic Health Evaluation, *ARDS* acute respiratory distress syndrome, *ECMO* extracorporeal membrane oxygenation

^a^In accordance with Berlin definition

^b^Oral prednisolone equivalent dosage > 5 mg/day or > 150 mg cumulative dose within 1 month before influenza infection; or regular treatment using other immunosuppressants within 1month before influenza infection

^c^Excluding those with end-stage renal disease receiving regular hemodialysis

*< 0.05

**0 < 0.01

***0 < 0.001

To further elucidate risk factors for PMV in influenza-associated ARDS patients, univariate and multivariate logistic regression analyses were conducted (Table 2). Ultimately, it was determined that in ARDS patients with influenza complications, BMI > 25, ECMO use, combined with bacterial pneumonia, and neuromuscular blockade exceeding 48 h were significant independent variables associated with PMV. To reduce the selection bias, we also compared the PMV or death before MV day 21 group to non-PMV group (Additional file 2: Figure S1). In PMV or death before MV day 21 group, they had higher APACHE II score, more patients with severe ARDS, more use of ECMO before MV day 7, more patients with bacterial pneumonia before MV day 7, more use of neuromuscular blockade over 48 h, more need of vasopressors, and more need of renal replacement therapy compared with survived non-PMV group (Additional file 1: Table S1). Moreover, in PMV or death before MV day 21 group, they had longer days of MV support, longer ICU and hospital days and higher in-hospital mortality compared with survived non-PMV group. After a multivariate regression analysis, higher APACHE II score, ECMO before MV day 7, combined with bacterial pneumonia before MV day 7, neuromuscular blockade over 48 h, and the need for vasopressor agents were associated with patients with PMV or death before MV day 21 (Additional file 1: Table S2).

**Table 2:** Risk factors for PMV in patients with influenza-related ARDS

	Univariate			Multivariate		
	Odds ratio	95% confidence interval	*p* value	Odds ratio	95% confidence interval	*p* value
Body mass index (kg/m2)	1.046	0.992 ~ 1.103	0.095			
BMI > 25 (kg/m2)	2.583	1.423 ~ 4.688	0.002	2.087	1.006 ~ 4.329	0.048
Cardiac disease	0.401	0.144 ~ 1.114	0.08			
Combined with bacterial pneumonia onset before D21	3.893	2.120 ~ 7.149	< 0.001	4.115	2.002 ~ 8.456	< 0.001
Bacteremia onset before D21	2.619	1.244 ~ 5.517	0.011	1.821	0.755 ~ 4.391	0.18
APACHE II score	1.038	1.003 ~ 1.075	0.036	1.014	0.97 ~ 1.060	0.55
ECMO	8.062	3.428 ~ 18.964	< 0.001	6.181	2.338 ~ 16.336	< 0.001
Prone	1.785	0.934 ~ 3.411	0.079			
Neuromuscular blockade > 48 h	3.985	2.149 ~ 7.389	< 0.001	2.800	1.334 ~ 5.879	0.007
Need for vasopressor agents	2.597	1.464 ~ 4.606	< 0.001	1.528	0.753 ~ 3.099	0.24
Acute kidney injury requiring renal replacement therapy ^a^	2.647	1.016 ~ 6.896	0.046	2.578	0.778 ~ 8.543	0.121

*ARDS* acute respiratory distress syndrome, *ECMO* extracorporeal membrane oxygenation, *APACHE II* Acute Physiology and Chronic Health Evaluation

^a^Excluding those with end-stage renal disease receiving regular hemodialysis

To reduce the survival bias, we compared the PMV or death between MV D7 and D21 group with non-PMV group in patients with MV use more than 7 days (Additional file 2: Figure S2). In PMV or death between MV day 8 and MV day 21 group, they had higher APACHE II score, more use of ECMO before MV day 7, more patients with bacterial pneumonia before MV day 7, more bacteremia before MV day 7, more use of neuromuscular blockade over 48 h, more need of vasopressors, and more need of renal replacement therapy compared with survived non-PMV group (Additional file 1: Table S3). We also observed longer MV days, longer ICU and hospital days and higher in-hospital mortality in PMV or death between MV day 8 and MV day 21 group compared with survived non-PMV group. In addition, ECMO before MV day 7, patients with bacterial pneumonia and use of neuromuscular blockade over 48 h were risk factors for PMV or death between MV day 8 and MV day 21 (Additional file 1: Table S4).

**Fig. 2:**
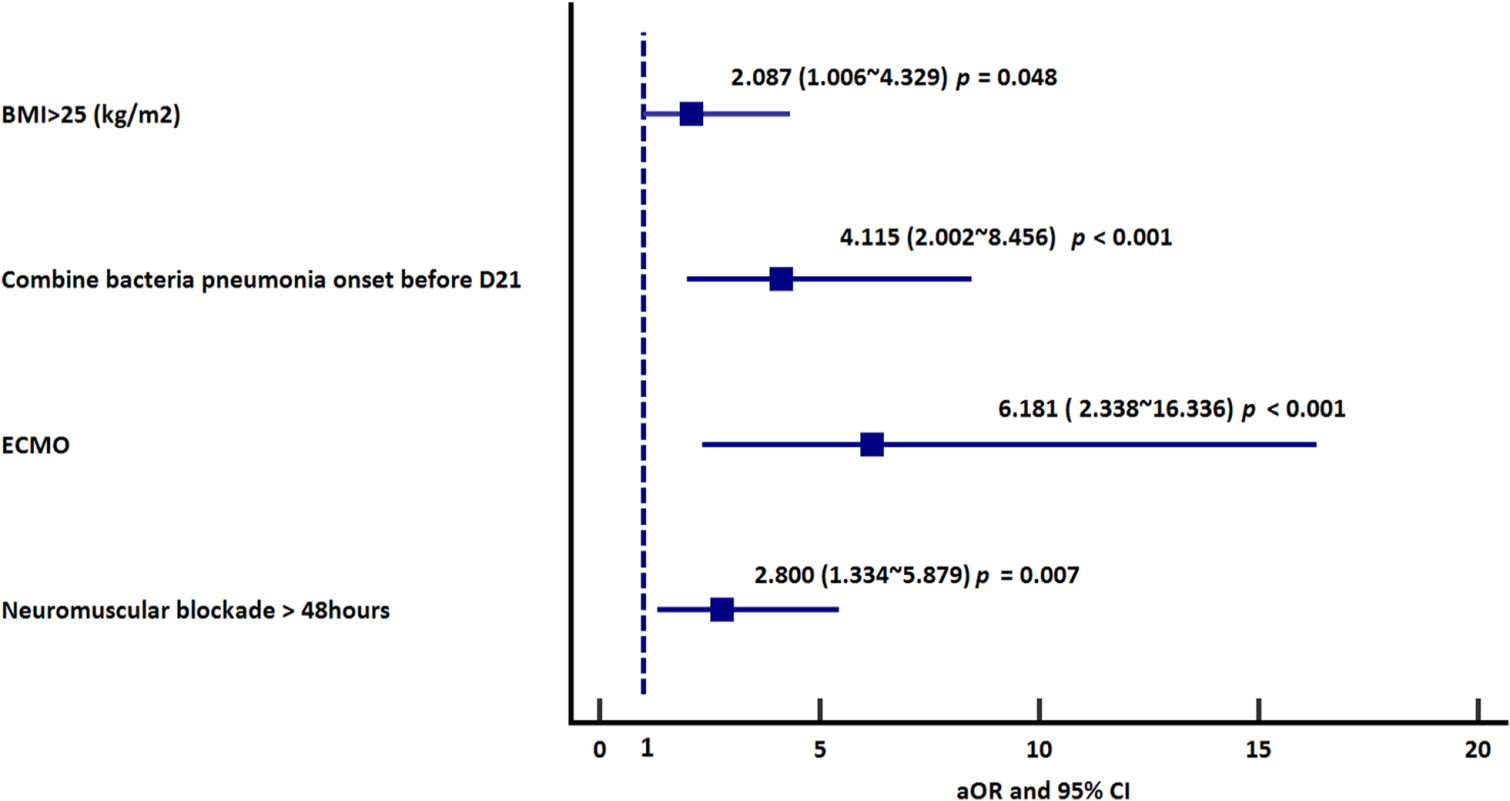
Factors associated with PMV. Forest plot of significant variables included in multivariable regression analysis. Odds ratios are reported with 95% confidence intervals

**Fig. 3:**
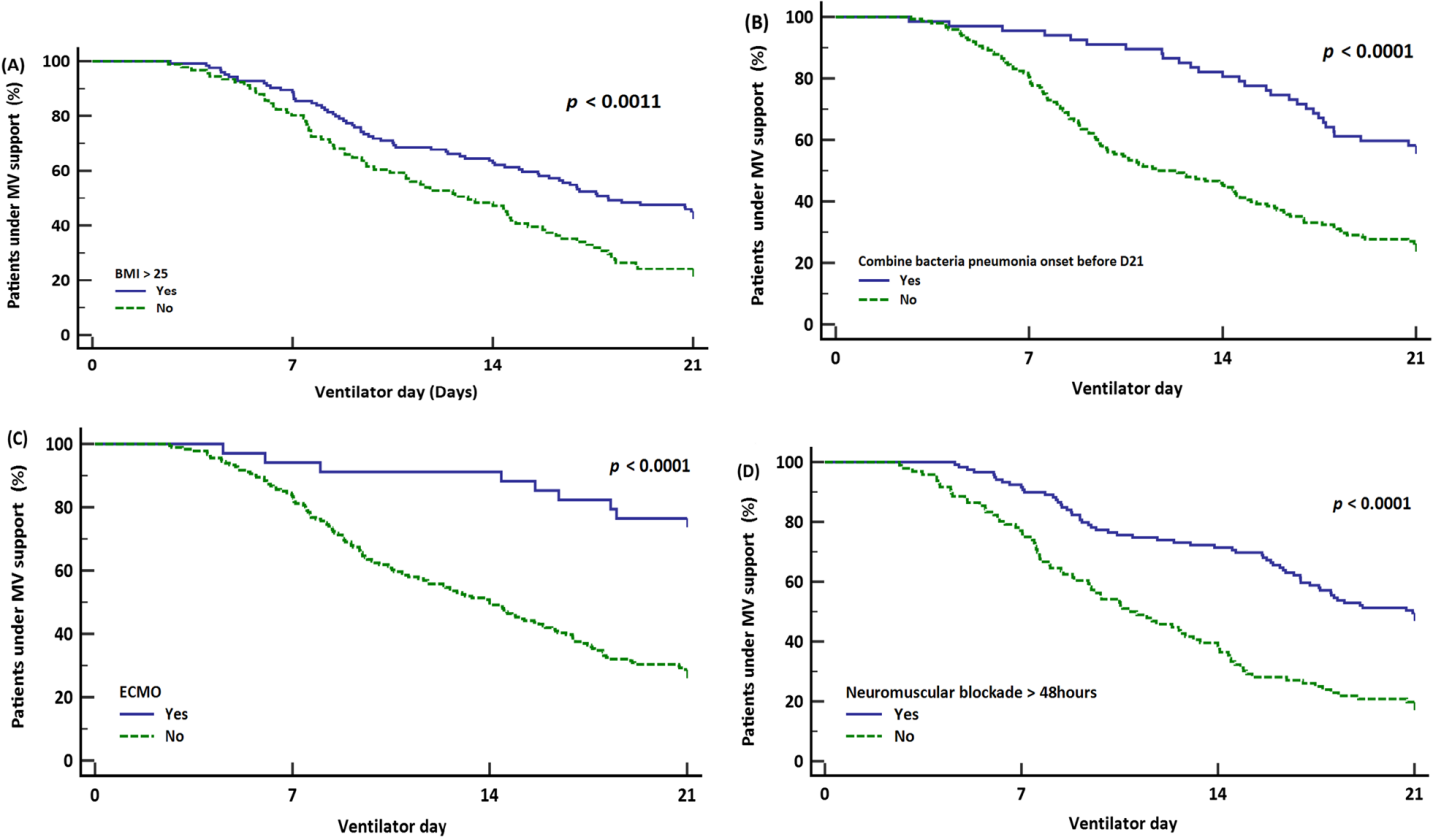
Kaplan‒Meier survival analysis for PMV risk factors. **A** In estimation for prolonged mechanical ventilation (PMV) occurrence in influenza-induced ARDS, stratification was conducted based on BMI > 25 (kg/m^2^). Individuals with BMI > 25 (kg/m^2^) exhibited a significantly higher prevalence of PMV. **B** In estimation of prolonged mechanical ventilation (PMV) occurrence in influenza-induced ARDS, stratification was performed based on the presence or absence of combined bacterial pneumonia within 21 days prior to ventilator support. Individuals with combined bacterial pneumonia showed a significantly higher incidence of PMV. **C** In estimation for prolonged mechanical ventilation (PMV) in influenza-induced ARDS, stratification was performed based on the presence or absence of ECMO utilization. Individuals receiving ECMO support demonstrated a significantly higher incidence of PMV. **D** In estimation for prolonged mechanical ventilation (PMV) occurrence in influenza-induced ARDS, stratification was performed based on the duration of neuromuscular blockade agent (NMBA) use exceeding 48 h. Individuals with NMBA use exceeding 48 h showed a significantly higher incidence of PMV

The results of binary logistic regression analysis—forest plot of variables independently associated with PMV are shown in Fig. 2. confirming that patients with BMI > 25 kg/m^2^, ECMO use, concomitant bacterial pneumonia, and neuromuscular blockade use over 48 h had substantial risks for PMV. In survival analysis, we found that BMI > 25 kg/m^2^, patients with bacterial pneumonia, ECMO and use of neuromuscular blockade more than 48 h were also associated with longer duration of MV support (Fig. 3A–D).

For secondary outcomes, we assessed 78 patients with Prolonged Mechanical Ventilation (PMV), analyzing risk factors associated with unsuccessful extubation in PMV patients (Table 3). The mean duration of mechanical ventilation was 39.24 (± 17.77) days. Out of these PMV patients, 44 were eventually successfully weaned from MV.

**Table 3:** Risk factors for weaning failure in PMV patients

	Univariate			Multivariate		
	Odds ratio	95% confidence interval	*p* value	Odds ratio	95% confidence interval	*p* value
ECMO	5.06	1.83 ~ 14.04	0.002	5.05	1.75 ~ 14.58	0.003
Bacteremia	3.92	1.30 ~ 11.83	0.015	3.91	1.20 ~ 12.69	0.023

*ECMO* extracorporeal membrane oxygenation

Thirty-four patients either died or required ongoing ventilator support after 21 days of ventilation. Following univariate and multivariate regression analyses in these two patient groups (Table 3), we confirmed that the use of ECMO support (OR: 5.05; 95% CI 1.75–14.58) and bacteremia (OR: 3.91; 95% CI 1.20–12.69) were risk factors associated with failed ventilator liberation after 21 days of ventilation.

Throughout the follow-up period, 181 patients achieved successful ventilator weaning, resulting in a ventilator weaning rate of 68.8%.

## Discussion

Patients with influenza related ARDS have independent risk factors to develop PMV, including BMI > 25, combined bacterial pneumonia, neuromuscular blockade use over 48 h during treatment, and ECMO support. Additionally, we found that risk factors for unsuccessful weaning after 21 days of ventilation are ECMO support and presence of bacteremia.

In our cohort, an average BMI > 25 (overweight and obese) was observed, with a higher prevalence of patients with BMI > 25 in the PMV group. The most recent studies have described that individuals with obesity who contract influenza are more likely to require mechanical ventilation and experience longer stays in the intensive care unit (ICU), along with an increased risk of mortality [10, 11]. One of clinical studies on ARDS have noted higher BMI in ARDS patients than in non-ARDS patients. Development of ARDS increases significantly with higher body weight [12]. A meta-analysis demonstrated a significant association between obesity in critically ill patients and prolonged mechanical ventilation duration [13]. Over the past two years, the literature has begun to highlight that the proportion of patients with BMI > 25 of COVID-19 is nearly twice that of those with influenza [14]. There was a trend towards a positive association between the BMI (normal weight, overweight and obesity) and the risk of serious events linked to COVID-19, with a marked increase from 8.1% to 20% and 30.6% respectively [15]. A COVID-19 study also indicated that patients with higher BMI spent more days on ventilators than those with normal weight, aligning with our cases and the results of this study [16].

The combination of influenza virus and bacterial pneumonia can exacerbate the severity of ARDS, infectious shock, and multiorgan failure. [17] In patients requiring hospitalization, bacterial pneumonia is more commonly seen in influenza than in COVID-19. [18, 19]. During influenza virus infection, changes in respiratory epithelial cells and host immune responses lead to exposure of the epithelial surface. As the infection progresses, bacteria can adhere, and respiratory bacteria can accumulate in the airway epithelial mucus, thereby promoting secondary bacterial pneumonia [20]. In the 2009 H1N1 pandemic, bacterial pneumonia as a pulmonary complication was associated with prolonged duration of mechanical ventilation. [21] In our cohort, the presence of bacterial pneumonia prolonged the duration of mechanical ventilation, and in another study conducted by our team, we found that severe influenza-related ARDS hospital-acquired lower respiratory tract infections are associated with prolonged mechanical ventilation and worse prognosis [22]. Another multicenter Italian study on COVID-19 patient weaning from ventilators also identified late-onset ventilator-associated pneumonia as one of the factors influencing ventilator liberation. [23]。In another study on COVID-19 with superinfections, bacteremia accounted for 47.4% of cases, and patients with secondary infections had a prolonged mechanical ventilation time of up to 37 days. [24] Bacteremia can progress to systemic inflammatory response syndrome (SIRS), sepsis, septic shock, and multiple organ dysfunction syndrome (MODS) [25, 26]. The occurrence of sepsis can affect diaphragmatic stability and lead to failed ventilator liberation. The presence of sepsis is associated with evident diaphragmatic weakness. [27–31] Currently, diaphragm dysfunction in critically ill patients is believed to occur primarily through two mechanisms: ventilator-induced diaphragmatic dysfunction (VIDD) [32] and sepsis-induced dysfunction. [33] Sepsis typically impairs oxygen consumption, increases anaerobic metabolism, and leads to metabolic acidosis. The need to compensate for acidemia increases ventilation requirements and may result in failed ventilator liberation [34].

In recent years, research has indicated that spontaneous ventilation can help to improve hypoxemia and lung compliance and reduce diaphragm atrophy in patients with acute respiratory distress syndrome (ARDS).

[35–37] However, spontaneous breathing can lead to elevated respiratory drive and vigorous inspiratory efforts, causing uneven pressure distribution and potentially resulting in patient self-inflicted lung injury (P-SILI) [38]. Multiple animal studies have demonstrated detrimental cycles, such as asynchrony with the ventilator, elevated transpulmonary pressure, and double triggering, which further worsen lung injury. [28, 39–41] A trial in 2010 reported improved 90-day survival rates in severe ARDS patients receiving neuromuscular blockade (NMBAs) [42], but a larger trial in 2019 contradicted these findings. [43] Therefore, use of NMBAs in ARDS patients remains controversial. Although current evidence does not support routine early use of neuromuscular blockade in all adult patients with moderate to severe ARDS, utilizing NMBAs in the early stages of ARDS to ensure good synchrony with the ventilator and promote lung-protective strategies remains a reasonable treatment option. There are formal guidelines that recommend continuous infusion of NMBAs for less 48 h [44] and evaluation daily by specialized physicians for ongoing use. [45, 46] Clinical data indicate that using neuromuscular blocking agents can reduce barotrauma and improve physiological and clinical outcomes. [47] However, there are potential adverse effects on diaphragmatic contractile function and delayed extubation. [48] Several studies and a meta-analysis on acute respiratory distress syndrome (ARDS) have confirmed that neuromuscular blocking agents (NMBAs) do not improve mortality rates, ventilator-free days (VFDs), or the duration of mechanical ventilation. [49–51]. However, it is important to note that the incongruences in research methodologies lead to an inability to reach a definitive consensus on this matter.

In contrast, our study findings indicate a significant impact of utilizing NMBAs for more than 48 h on the extension of mechanical ventilation duration, known as prolonged mechanical ventilation (PMV).

ECMO provides circulatory or respiratory support in cases of refractory cardiogenic shock or ARDS. Multiple studies have indicated that ECMO is feasible and effective for ARDS patients caused by H1N1 infection in 2009[52–54].One study assessing ECMO-related mortality risk reported that the mortality rate for influenza-induced ARDS patients receiving ECMO support was the lowest observed thus far, despite an average duration of mechanical ventilation support of up to 40 days. [55]。

In a multicenter retrospective study conducted in Italy, COVID-19 and influenza-related ARDS receiving ECMO patients had longer durations of invasive mechanical ventilation than influenza patients, with durations of 33 days and 25 days[56], respectively. However, the mortality rate during COVID-19 was higher than that during the 2009 H1N1 period, potentially due to more frequent use of noninvasive ventilation (HFNC) forms before endotracheal intubation, leading to more severe self-inflicted lung injury. [38, 57]。In studies involving ECMO usage for H1N1 influenza patients, the duration of mechanical ventilation before ECMO initiation was identified as an important prognostic factor. [54] A multicenter study conducted in COVID-19 patients yielded similar results. [58] Numerous international multicenter studies have substantiated the elevated mortality rates associated with ARDS, which are estimated to be approximately 40% [59–62]. In another study focusing on the association of higher tidal volumes with increased mortality conducted by our group, a 30-day mortality rate of 23.2% was observed [63], but the estimated mortality rate for patients after PMV increased to 39.7%. This similarity with large international studies confirms the association between PMV and a higher mortality rate.

Our study has several limitations. First, it is a retrospective study, which may have resulted in missing statistical and medical data, leading to variations. Second, there was a lack of consistency in the treatment strategies for influenza-related ARDS among the different study sites. The treatment policies of participating centers were not standardized, which increased potential confounding factors. Last, our study focused on patients with ARDS caused by influenza. Therefore, whether the findings apply to ARDS caused by other factors needs to be confirmed through future, more rigorous prospective clinical studies. Despite these limitations, to the best of our knowledge, this is the first multicenter study that elucidates risk factors associated with prolonged mechanical ventilation in patients with ARDS caused by influenza. Our study may aid clinicians with regard to treatment directions and decision-making for critically ill patients.

## Conclusion

Patients with influenza-related ARDS and subsequent PMV have a high mortality rate. The present study identified several independent predictors of prolonged mechanical ventilation (PMV) in influenza-associated ARDS. These predictors included BMI > 25, combine bacterial pneumonia, the use of neuromuscular blockers more than 48 h during ICU stay, and ECMO support. Additionally, unsuccessfully weaning from mechanical ventilation was independently associated with ECMO support during hospitalization or development of bacteremia.

### Supplementary Information

**Additional file 1: Table S1.** Characteristics of the 263 subjects with influenza-related ARDS. **Table S2. **Risk factors for PMV or death before MV D21 in subjects with influenza-related ARDS. **Table S3.**Characteristics of the subjects with influenza-related ARDS and MV use > 7 days. **Table S4. R**isk factors for PMV in patients with influenza-related ARDS.

**Additional file 2: Figure S1.** Flow chart of the study. a.Virology-proven methods include the rapid influenza diagnostic test, reverse transcription-polymerase chain reaction and virus culture. ARDS, acute respiratory distress syndrome; ICU, intensive care unit; MV, mechanical ventilator. **Figure S2.**

## Data Availability

The data presented in this study are available on request from the corresponding author.
