# Supplementary material for: Risk factors for prolonged mechanical ventilation in critically ill patients with influenza-related acute respiratory distress syndrome
Source: Respir Res. 2024 Jan 4;25:9. doi: 10.1186/s12931-023-02648-3 (PMC10765923; doi:10.1186/s12931-023-02648-3)
Supplement: Supplementary file 1 — Additional file 1: Table S1. Characteristics of the 263 subjects with influenza-related ARDS. Table S2. Risk factors for PMV or death before MV D21 in subjects with influenza-related ARDS. Table S3.Characteristics of the subjects with influenza-related ARDS and MV use > 7 days. Table S4. Risk factors for PMV in patients with influenza-related ARDS. [file 12931_2023_2648_MOESM1_ESM.docx]

**Table S1.** Characteristics of the 263 subjects with influenza-related ARDS

|  | All patients | Prolonged mechanical ventilation (PMV)  or death before MV D21 | |  | Weaning in PMV | |  |
| --- | --- | --- | --- | --- | --- | --- | --- |
|  |  | **Yes** | **No** | ***p* Value** | **Success** | **Failure** | ***p* Value** |
| Characteristics | **(n=263)** | **(n=126)** | **(n=137)** |  | **(n=44)** | **(n=34)** |  |
| Baseline data | | | | | | | |
| Age (years) | **59.78(±14.63)** | **59.68(±13.27)** | **59.88(±15.83)** | **0.92** | **59.25(±12.12)** | **56.88(±11.27)** | **038** |
| Male sex | **166(63.1%)** | **82(65.1%)** | **84(61.3%)** | **0.53** | **31(70.5%)** | **19(55.9%)** | **0.18** |
| Body mass index BMI (kg/m2) | **25.19(±5.58)** | **24.92(±4.93)** | **25.44(±6.12)** | **0.45** | **26.65(±4.19)** | **27.09(±5.48)** | **0.68** |
| BMI>25 (kg/m2) | **131(49.8%)** | **64(50.8%)** | **67(48.9%)** | **0.76** | **23(67.6%)** | **33(75%)** | **0.47** |
| Malignancy | **35(13.3%)** | **21(16.7%)** | **14(10.2%)** | **0.12** | **7(15.9%)** | **5(14.7%)** | **0.88** |
| Type II diabetes mellitus | **76(28.9%)** | **35(27.8%)** | **41(29.9%)** | **0.70** | **11 (25%)** | **11(32.4%)** | **0.47** |
| Cerebrovascular disease | **22(8.4%)** | **12(9.5%)** | **10(7.3%)** | **0.52** | **4** | **0** | **0.13** |
| Liver disease | **34(12.9%)** | **21(16.7%)** | **13(9.5%)** | **0.08** | **4(11.8%)** | **5(11.4%)** | **1.0** |
| Cardiac disease | **25(9.5%)** | **8(6.3%)** | **17(12.4%)** | **0.09** | **3(8.8%)** | **2(4.5%)** | **0.65** |
| Hypertension | **108(41.1%)** | **50(39.7%)** | **58(42.3%)** | **0.66** | **19(43.2%)** | **16(47.1%)** | **0.73** |
| Immunosuppressant^b^  use before  influenza infection | **26(9.9%)** | **13(10.3%)** | **13(9.5%)** | **0.82** | **1(2.3%)** | **3(8.8%)** | **0.31** |
| Autoimmune disease | **18(6.8%)** | **10(7.9%)** | **8(5.8%)** | **0.50** | **2(4.5%)** | **4(11.8%)** | **0.40** |
| End-stage renal disease | **15(5.7%)** | **8(6.3%)** | **7(5.1%)** | **0.67** | **4(9.1%)** | **3(8.8%)** | **1.0** |
| Severity score | | | | | | | |
| APACHE II score | **23.76±8.53** | **25.99±8.75** | **21.7±7.80** | **<0.001** | **23.30±8.74** | **25.24(±8.23)** | **0.32** |
| ARDS ^a^ Severity |  |  |  | **0.04** |  |  | **0.76** |
| Severe | **156(59.3%)** | **83(65.9%)** | **73(53.3%)** |  | **27(61.4%)** | **22(64.7%)** |  |
| Mild to moderate | **107(40.7%)** | **43(34.1%)** | **64(46.7%)** |  | **17(38.6%)** | **12(35.3%)** |  |
| Treatments and clinical outcome | | | | | | | |
| Prone | **61(23.2%)** | **35(27.8%)** | **26(19%)** | **0.09** | **14(31.8%)** | **9(26.5%)** | **0.61** |
| ECMO before MV D7 | **47(17.9%)** | **39(31%)** | **8(5.8%)** | **<0.001** | **8(18.2%)** | **18(52.9%)** | **<0.01** |
| Combined with bacterial pneumonia onset before MV D7 | **57(21.7%)** | **35(27.8%)** | **22(16.1%)** | **0.021** | **26(59.1%)** | **22(64.7%)** | **0.61** |
| Bacteremia onset before D7 | **39(14.8%)** | **24(19%)** | **15(10.9%)** | **0.07** | **6(13.6%)** | **13(38.2%)** | **0.012** |
| Steroid user | **162(61.6%)** | **81(64.3%)** | **81(59.1%)** | **0.39** | **23(52.3%)** | **23(67.6%)** | **0.17** |
| Sedation | **193(73.4%)** | **94(74.6%)** | **99(72.3%)** | **0.67** | **32(72.7%)** | **28(82.4%)** | **0.32** |
| Neuromuscular blockade > 48 h | **148(56.3%)** | **88(69.8%)** | **60(43.8%)** | **<0.001** | **32(72.7%)** | **27(79.4%)** | **0.5** |
| Need for vasopressor agents | **140(53.2%)** | **86(68.3%)** | **54(39.4%)** | **<0.001** | **25(56.8%)** | **24(70.6%)** | **0.21** |
| Renal replacement therapy ^c^ | **31(11.8%)** | **23(18.3%)** | **8(5.8%)** | **0.002** | **6(13.6%)** | **5(14.7%)** | **1.0** |
| Ventilator-duration (days) | **18.97(±16.89)** | **27.75(±20.60)** | **10.90(±4.77)** | **<0.001** | **36.12(±14.60)** | **43.29(±20.72)** | **0.09** |
| ICU stay (days) | **19.82(±17.29)** | **26.96(±21.98)** | **13.22(±6.24)** | **<0.001** | **36(±19.22)** | **41(±22.34)** | **0.29** |
| Hospital-stay (days) | **32.61(±27.04)** | **37.45(±33.63)** | **28.11(±18.00)** | **<0.001** | **58.90(±29.70)** | **47.92(±35.77)** | **0.14** |
| In hospital Mortality | **90(34.2%)** | **79(62.7%%)** | **11(8.0%)** | **<0.001** | **5(11.4%)** | **26(76.5%)** | **<0.001** |
| Data are presented as the mean ± standard deviation and number (%).  ^a^ In accordance with Berlin definition  ^b^ Oral prednisolone equivalent dosage > 5 mg/day or >150 mg cumulative dose within 1 month before influenza infection; or regular treatment using other immunosuppressants within 1month before influenza infection  ^c^ Excluding those with end-stage renal disease receiving regular hemodialysis  APACHE II, Acute Physiology and Chronic Health Evaluation.  ARDS, acute respiratory distress syndrome.  ECMO, extracorporeal membrane oxygenation | | | | | | | |

**Table S2. Risk factors for PMV or death before MV D21 in subjects with influenza-related ARDS**

|  | **Univariate** | | | **Multivariate** | | |
| --- | --- | --- | --- | --- | --- | --- |
|  | **Odds ratio** | **95%**  **confidence interval** | ***p* value** | **Odds ratio** | **95%**  **confidence interval** | ***p* value** |
| **Liver disease** | **1.91** | **0.91-3.99** | **0.09** |  |  |  |
| **Cardiac disease** | **0.48** | **0.20-1.15** | **0.10** |  |  |  |
| **APACHE II score** | **1.07** | **1.03-1.1** | **<0.001** | **1.05** | **1.01-1.08** | **0.01** |
| **ARDS ^a^ Severity** | **0.59** | **0.36-0.97** | **0.04** | **1.17** | **0.64-2.13** | **0.61** |
| **Prone** | **1.64** | **0.92-2.93** | **0.09** |  |  |  |
| **ECMO before MV D7** | **7.23** | **3.22-16.21** | **<0.001** | **6.03** | **2.56-14.22** | **<0.001** |
| **Combined with bacterial pneumonia onset before MV D7** | **2.01** | **1.10-3.66** | **0.02** | **2.02** | **1.02-4.00** | **0.044** |
| **Bacteremia onset before D7** | **1.91** | **0.95-3.84** | **0.07** |  |  |  |
| **Neuromuscular blockade > 48 hours** | **2.97** | **1.79-4.94** | **<0.001** | **2.37** | **1.28-4.39** | **0.01** |
| **Need for vasopressor agents** | **3.31** | **1.99-5.49** | **<0.001** | **1.95** | **1.08-3.53** | **0.03** |
| **Acute kidney injury requiring renal replacement therapy ^a^** | **3.60** | **1.55-8.38** | **<0.001** | **2.13** | **0.80-5.64** | **0.13** |
| **ARDS, acute respiratory distress syndrome.**  **ECMO, extracorporeal membrane oxygenation.**  **APACHE II, Acute Physiology and Chronic Health Evaluation.**  **^a^ Excluding those with end-stage renal disease receiving regular hemodialysis** | | | | | | |

**Table S3. Characteristics of the subjects with influenza-related ARDS and MV use > 7 days**

|  | All patients | MV use > 7days  (N=213) | |  | Weaning in PMV | |  |
| --- | --- | --- | --- | --- | --- | --- | --- |
|  |  | **PMV or death between MV D7 and D21 group** | **Non-PMV group** | ***p* Value** | **Success** | **Failure** | ***p* Value** |
| Characteristics | **(n=213)** | **(n=106)** | **(n=107)** |  | **(n=44)** | **(n=34)** |  |
| Baseline data | | | | | | | |
| Age (years) | **59.47(±14.22)** | **59.08(±13.19)** | **59.86(±15.22)** | **0.692** | **59.25(±12.12)** | **56.88(±11.27)** | **038** |
| Male sex | **132(62%)** | **69(65.1%)** | **63(58.9%)** | **0.35** | **31(70.5%)** | **19(55.9%)** | **0.18** |
| Body mass index BMI (kg/m2) | **25.50(±5.07)** | **25.41(±4.98)** | **25.39(±5.12)** | **0.978** | **26.65(±4.19)** | **27.09(±5.48)** | **0.68** |
| BMI>25 (kg/m2) |  | **59(55.7%)** | **53(49.5%)** | **0.371** | **23(67.6%)** | **33(75%)** | **0.47** |
| Malignancy | **29(13.6%)** | **18(17%)** | **11(10.3%)** | **0.154** | **7(15.9%)** | **5(14.7%)** | **0.88** |
| Type II diabetes mellitus | **61(28.6%)** | **28(26.4%)** | **33(30.8%)** | **0.475** | **11 (25%)** | **11(32.4%)** | **0.47** |
| Cerebrovascular disease | **14(6.6%)** | **7(6.6%)** | **7(6.5%)** | **0.985** | **4** | **0** | **0.13** |
| Liver disease | **28(13.1%)** | **17(16%)** | **11(10.3%)** | **0.214** | **4(11.8%)** | **5(11.4%)** | **1.0** |
| Cardiac disease | **16(7.5%)** | **6(5.7%)** | **10(9.3%)** | **0.308** | **3(8.8%)** | **2(4.5%)** | **0.65** |
| Hypertension | **88(41.3%)** | **43(40.6%)** | **45(42.1%)** | **0.825** | **19(43.2%)** | **16(47.1%)** | **0.73** |
| Immunosuppressant^b^  use before  influenza infection | **25(11.7%)** | **11(10.4%)** | **14(13.1%)** | **0.539** | **1(2.3%)** | **3(8.8%)** | **0.31** |
| Autoimmune disease | **16(7.5%)** | **7(6.6%)** | **9(8.4%)** | **0.617** | **2(4.5%)** | **4(11.8%)** | **0.40** |
| End-stage renal disease | **12(5.6%)** | **7(6.6%)** | **5(4.7%)** | **0.541** | **4(9.1%)** | **3(8.8%)** | **1.0** |
| Severity scores | | | | | | | |
| APACHE II score | **23.15±8.10** | **24.43±8.20** | **21.88±7.83** | **0.021** | **23.30±8.74** | **25.24(±8.23)** | **0.32** |
| ARDS ^a^ Severity |  |  |  | **0.613** |  |  | **0.76** |
| Severe | **129(60.6%)** | **66(62.3%)** | **63(58.9%)** |  | **27(61.4%)** | **22(64.7%)** |  |
| Mild to moderate | **84(39.4%)** | **40(37.7%)** | **44(41.1%)** |  | **17(38.6%)** | **12(35.3%)** |  |
| Treatments and clinical outcome | | | | | | | |
| Prone | **54(25.4%)** | **31(29.2%)** | **23(21.5%)** | **0.194** | **14(31.8%)** | **9(26.5%)** | **0.61** |
| ECMO before MV D7 | **41(19.2%)** | **35(33%)** | **6(5.6%)** | **<0.001** | **8(18.2%)** | **18(52.9%)^＊＊^** | **<0.01** |
| Combined with bacterial pneumonia onset before MV D7 | **51(23.9%)** | **32(30.2%)** | **19(17.8%)** | **0.034** | **26(59.1%)** | **22(64.7%)** | **0.61** |
| Bacteremia onset before D7 | **34(16%)** | **24(70.6%)** | **10(9.3%)** | **0.008** | **6(13.6%)** | **13(38.2%)^＊^** | **0.012** |
| Steroid user | **131(61.5%)** | **67(63.2%)** | **64(59.8%)** | **0.611** | **23(52.3%)** | **23(67.6%)** | **0.17** |
| Sedation | **165(77.5%)** | **83(78.3%)** | **82(76.6%)** | **0.771** | **32(72.7%)** | **28(82.4%)** | **0.32** |
| Neuromuscular blockade > 48 h | **129(60.6%)** | **77(72.6%)** | **52(48.6%)** | **<0.001** | **32(72.7%)** | **27(79.4%)** | **0.5** |
| Need for vasopressor agents | **120(56.3%)** | **71(67%)** | **49(45.8%)** | **<0.002** | **25(56.8%)** | **24(70.6%)** | **0.21** |
| Renal replacement therapy before D7 ^c^ | **23(10.8%)** | **17(16%)** | **6(5.6%)** | **0.014** | **6(13.6%)** | **5(14.7%)** | **1.0** |
| Ventilator-duration (days) | **22.40 (±17.1)** | **32.41(±19.13)** | **12.50(±4.14)** | **<0.001** | **36.12(±14.60)** | **43.29(±20.72)** | **0.09** |
| ICU stay (days) | **23.17 (±17.54)** | **31.59(±20.93)** | **14.75(±6.13)** | **<0.001** | **36(±19.22)** | **41(±22.34)** | **0.29** |
| Hospital-stay (days) | **36.65 (±28.89)** | **43.72(±33.07)** | **29.58(±18.18)** | **<0.001** | **58.90(±29.70)** | **47.92(±35.77)** | **0.14** |
| In hospital Mortality | **64(30%)** | **54(50.9%)** | **10(9.3%)** | **<0.001** | **5(11.4%)** | **26(76.5%)^＊＊＊^** | **<0.001** |

Data are presented as the mean ± standard deviation and number (%).

^a^ In accordance with Berlin definition

^b^ Oral prednisolone equivalent dosage > 5 mg/day or >150 mg cumulative dose within 1 month before influenza infection; or regular treatment using other immunosuppressants within 1month before influenza infection

^c^ Excluding those with end-stage renal disease receiving regular hemodialysis

APACHE II, Acute Physiology and Chronic Health Evaluation.

ARDS, acute respiratory distress syndrome.

ECMO, extracorporeal membrane oxygenation

**Table S4. Risk factors for PMV in patients with influenza-related ARDS.**

|  | **Univariate** | | | **Multivariate** | | |
| --- | --- | --- | --- | --- | --- | --- |
|  | **Odds ratio** | **95%**  **confidence interval** | ***p* value** | **Odds ratio** | **95%**  **confidence interval** | ***p* value** |
| **APACHE II score** | **1.04** | **1.01-1.08** | **0.02** | **1.01** | **0.97-1.05** | **0.56** |
| **ECMO before MV D7** | **8.30** | **3.32-20.77** | **<0.001** | **6.31** | **2.43-16.43** | **<0.001** |
| **Combined with bacterial pneumonia onset before MV D7** | **2.00** | **1.05-3.82** | **0.04** | **2.19** | **1.06-4.52** | **0.03** |
| **Bacteremia onset before D7** | **2.84** | **1.2.8-6.28** | **0.01** | **2.24** | **0.93-5.39** | **0.07** |
| **Neuromuscular blockade > 48 hours** | **2.81** | **1.59-4.97** | **<0.001** | **2.22** | **1.15-4.27** | **0.02** |
| **Need for vasopressor agents** | **2.40** | **1.38-4.18** | **<0.01** | **1.57** | **0.83-2.99** | **0.17** |
| **Acute kidney injury requiring renal replacement therapy before D7 ^a^** | **3.22** | **1.22-8.51** | **0.02** | **2.28** | **0.76-6.89** | **0.14** |

**APACHE II, Acute Physiology and Chronic Health Evaluation.**

**ECMO, extracorporeal membrane oxygenation.**

**^a^ Excluding those with end-stage renal disease receiving regular hemodialysis**
