# Supplementary material for: Risk factors for prolonged mechanical ventilation in critically ill patients with influenza-related acute respiratory distress syndrome
Source: Respir Res. 2024 Jan 4;25:9. doi: 10.1186/s12931-023-02648-3 (PMC10765923; doi:10.1186/s12931-023-02648-3)
Supplement: Supplementary file 2 — Additional file 2: Figure S1. Flow chart of the study. a.Virology-proven methods include the rapid influenza diagnostic test, reverse transcription-polymerase chain reaction and virus culture. ARDS, acute respiratory distress syndrome; ICU, intensive care unit; MV, mechanical ventilator. Figure S2. [file 12931_2023_2648_MOESM2_ESM.pptx]

## Slide 1
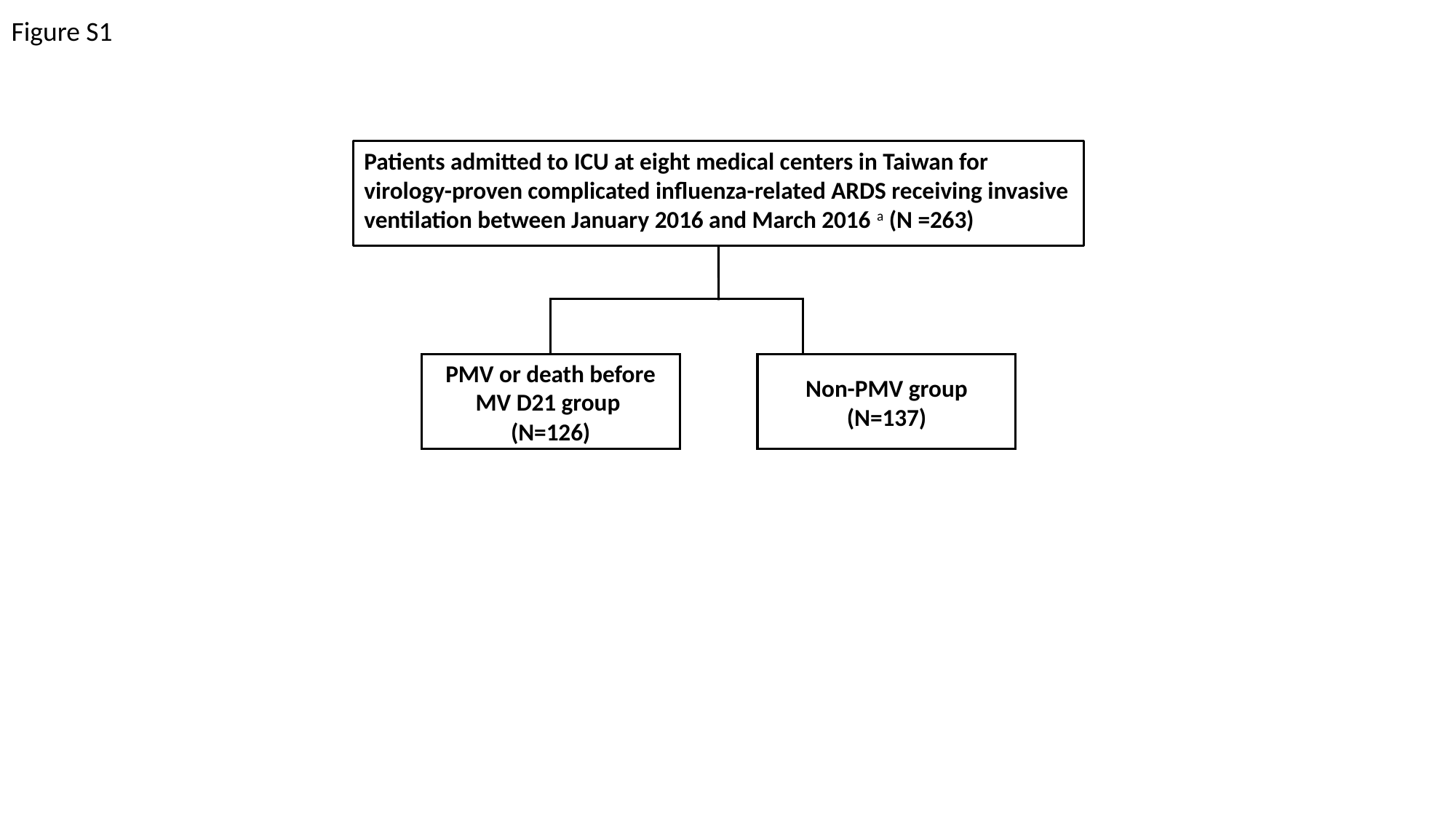

Figure S1
Patients admitted to ICU at eight medical centers in Taiwan for virology-proven complicated influenza-related ARDS receiving invasive ventilation between January 2016 and March 2016 a (N =263)
Non-PMV group
(N=137)
PMV or death before MV D21 group
(N=126)

## Slide 2
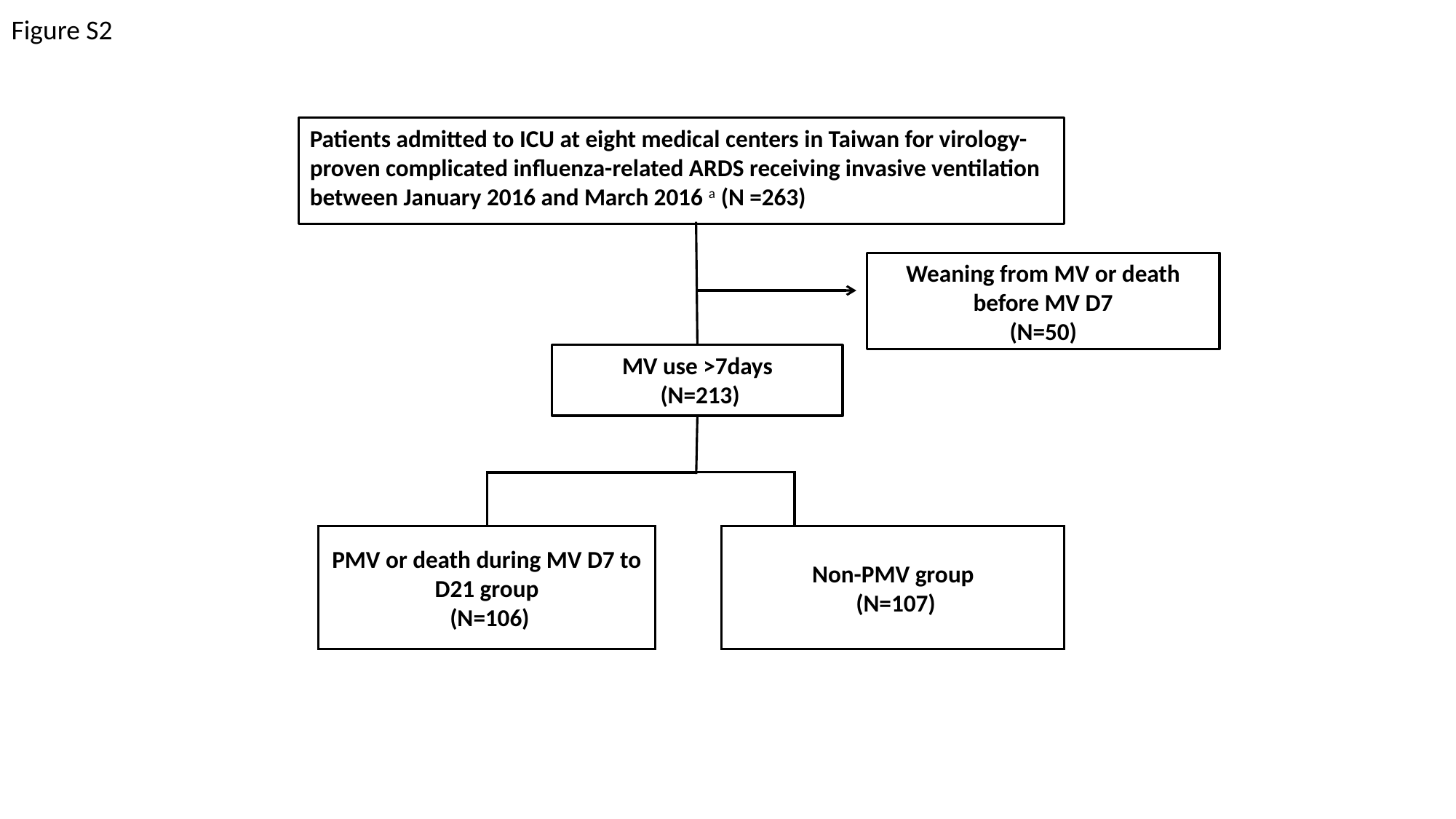

Figure S2
Patients admitted to ICU at eight medical centers in Taiwan for virology-proven complicated influenza-related ARDS receiving invasive ventilation between January 2016 and March 2016 a (N =263)
Weaning from MV or death before MV D7
(N=50)
MV use >7days
 (N=213)
Non-PMV group
 (N=107)
PMV or death during MV D7 to D21 group
 (N=106)
